# Classes of low-frequency earthquakes based on inter-time distribution reveal a precursor event for the 2011 Great Tohoku Earthquake

**DOI:** 10.1038/s41598-019-45765-0

**Published:** 2019-06-27

**Authors:** Tomoki Tokuda, Hirohiko Shimada

**Affiliations:** 1 Okinawa Institute of Science and Technology Graduate University, Onna, Okinawa 904-0495 Japan; 2 Department of Integrated Science and Technology, National Institute of Technology, Tsuyama College, 624-1 Numa, Tsuyama, Okayama, 708-8509 Japan

**Keywords:** Seismology, Applied mathematics

## Abstract

Recently, slow earthquakes (slow EQ) have received much attention relative to understanding the mechanisms underlying large earthquakes and to detecting their precursors. Low-frequency earthquakes (LFE) are a specific type of slow EQ. In the present paper, we reveal the relevance of LFEs to the 11 March 2011 Great Tohoku Earthquake (Tohoku-oki EQ) by means of cluster analysis. We classified LFEs in northern Japan in a data-driven manner, based on inter-time, the time interval between neighboring LFEs occurring within 10 km. We found that there are four classes of LFE that are characterized by median inter-times of 24 seconds, 27 minutes, 2.0 days, and 35 days, respectively. Remarkably, in examining the relevance of these classes to the Tohoku-oki EQ, we found that activity in the shortest inter-time class (median 24 seconds) diminished significantly at least three months before the Tohoku-oki EQ, and became completely quiescent 30 days before the event (p-value = 0.00014). Further statistical analysis implies that this class, together with a similar class of volcanic tremor, may have served as a precursor of the Tohoku-oki EQ. We discuss a generative model for these classes of LFE, in which the shortest inter-time class is characterized by a generalized gamma distribution with the product of shape parameters *v*κ = 1:54 in the domain of inter-time close to zero. We give a possible geodetic interpretation for the relevance of LFE to the Tohoku-oki EQ.

## Introduction

Detecting and identifying precursors to large earthquakes is essential in order to mitigate the devastating damage of earthquakes. In relation to the 11 March 2011 Great Tohoku Earthquake (Tohoku-oki EQ, hereafter) *M*_*w*_ 9.0, several precursors were reported retrospectively. With a long-term perspective, seismic quiescence was observed near the epicenter 23 years prior to Tohoku-oki EQ^1^. Also, strong correlations were observed between tidally induced stresses and earthquake occurrence times near the epicenter ten years before it occurred^2^. Further, an anomaly in the *b*-value of the Gutenberg-Richter law was reported near the epicenter five years before^3^. In the short term, it was observed that earthquake activities moved toward the epicenter one month before^4^. Regarding non-seismic phenomena, positive anomalies of ionospheric total electron content (TEC) were detected around the focal region 40 minutes before^5^. Further, anomalous changes of groundwater levels and Radon concentrations were reported three months before^6,7^. All these precursors are important not only for prediction of large earthquakes, but also for a better understanding of their underlying mechanisms. In the present study, we examine the relevance of slow earthquakes (slow EQ) to prediction of major events, focusing in particular on low-frequency earthquakes (LFE).

Slow EQs are low-frequency phenomena, distinguished from regular earthquakes, with lower dominant frequencies ranging from several Hz to inverse of several years^8^. Slow EQs include several subtypes such as LFEs, very low-frequency earthquakes, short-term slow-slip events, and long-term slow-slip events, depending on the range of dominant frequencies. Recently, such earthquakes have gained much attention both for better understanding the underlying mechanisms of earthquakes and for identifying precursors of large earthquakes^9–11^. Indeed, anomalous occurrences of slow EQs have been reported prior to large earthquakes^12^. In case of the Tohoku-oki EQ, it is inferred that slow-slip events occurred over a period of one month prior to the megathrust EQ^13^. It is argued that these slow events increased shear stress across a wide swath of the offshore of Tohoku region, which eventually triggered the Tohoku-oki EQ. In the Parkfield EQ, an anomalous change in occurrences of slow tremors was observed near the hypocenter three months before the earthquake^14^.

Slow EQs are commonly categorized into two categories: aseismic (geodetic) events and seismic events. The former include slow-slip events during short or long periods of time, while the latter include single low-frequency earthquakes (LFEs) and tremors comprising a number of LFEs. Despite various manifestations, slow EQs are basically caused by shear slips, the same as regular earthquakes^15^. However, slow EQs follow a different scaling law from regular earthquakes in seismic moment and characteristic duration. This suggests a longer duration of slow EQs for a given seismic moment than conventional earthquakes, though their mechanism is not well understood. The association between slow EQs and large earthquakes may be that slow EQs work as stress meters both in the long- and short-term. On one hand, long-term, slow-slip events reflect dominant changes in strains, which enable one to evaluate precise accumulation of strains^8^. On the other hand, short-term, slow-slip events or tremors may reflect nucleation processes leading to large earthquakes^4,16^. These assumptions suggest the importance of slow EQs as precursors for large EQs, both long- and short-term.

Some of the most compelling studies have focused on slow EQs near the epicenters of major earthquakes^4,13^. In particular, it was suggested that a nucleation process (that accelerates slow-slip movement, finally triggering a large earthquake) in the form of slow-slip events occurring near the epicenter. However, if slow EQs work as a true stress indicator, we would expect that some slow EQ anomaly might also occur even distant from the epicenter, because changes of stress prior to a large EQ may extend across a wide area of a continental plate. For instance, anomalous strain changes were identified in a borehole located in the Oshika Peninsula 150 km away from the epicenter before the Tohoku-oki EQ^13^. Furthermore, we would expect that such anomalous slow EQs, which occur away from the epicenter along the downdip edge of the megathrust seismogenic zone, might take the form of LFEs^8^. To the best of our knowledge, however, there has been no study that tried to shed light on LFEs far from the epicenter of a large EQ. The overall goal of the present study is to examine behavior of LFEs away from the epicenter of the Tohoku-oki EQ.

**Figure 1:**
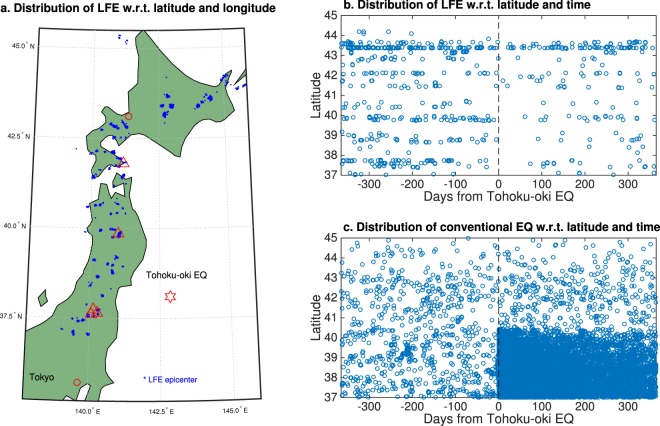
Distribution of LFE and conventional EQ. Panel a. Latitude and longitude distribution. Locations of epicenters of LFE (blue dots) and five volcanoes (triangles): Esan, Iwatesan, Azumayama, Adatarayama, and Bandaisan from North to South. The red hexagram denotes the epicenter of the Tohoku-oki EQ. Panel b. Latitude and temporal distribution of LFE. Panel c. Latitude and temporal distribution of conventional EQ with magnitude greater or equal to three. An apparent onset of large seismicity in conventional EQ just before the Tohoku-oki EQ is due to the occurrence of the foreshock with *M*_*w*_ = 7.3 near the epicenter of the Tohoku-oki EQ two days before. In all panels, we focused on earthquakes in latitudes between 37°N and 45°N, and in longitudes between 139°E and 146°E.

Toward this end, we studied LFEs that occurred in northern Japan, several hundred kilometers from the epicenter of the Tohoku-oki EQ (Fig. 1a). As is well-known, it can be observed that LFEs occurred in specific areas in a cluster-like manner. Note that the detection of these LFEs is not based on an area-specific seismic network, but on an extensive dense network of highly short-period instruments installed by National Research Institute for Earth Science and Disaster Prevention (NIED)^17^. This means, in the areas where no LFEs were observed, the extensive seismic network did not detect a LFE. The underlying physical mechanism of LFE in this region is not fully understood^17,18^. However, it is inferred from the analysis of velocities of P- and S-waves that LFEs in this region are related to aqueous fluids supplied by the subducted slab^19^. These fluids, originating from the mantle wedge in the continental plate, move up, resulting in accumulation of a large amount of melt below the Moho discontinuity. It is speculated that a sudden movement of such fluids near the Moho discontinuity may cause LFEs. Further, it is observed that the low seismicity of LFEs in this region seems synchronized with that of earthquakes in the Wadati-Benioff zone or the shallow inland seismicity, which implies that generation of LFEs may be related to changes in tectonic stress fields over a wide area^18^. With regard to the Tohoku-oki EQ, it is reported that in a spatial analysis, LFEs became generally less active after the Tohoku-oki EQ^20^, which contrasts with activation of conventional EQs in this region (Fig. 1b,c). However, such a spatial analysis does not reveal by itself whether such a change in seismicity occurred before the Tohoku-oki EQ.

In the present paper, to clarify this point, we employ a different approach to analyze LFEs, focusing on the inter-time distribution, the time between consecutive events, rather than on their spatial distribution. For conventional EQs, the distribution of inter-time has recently attracted attention because the inter-time with a cutoff magnitude (from 5 to 6.5) seems to entail a universal law in the form of a generalized gamma distribution^21–24^. With respect to the Tohoku-oki EQ, the parameters of such a generalized gamma distribution in the Tohoku region changed (as seen later in Table 2). In the context of LFEs, it is not obvious what distribution the inter-time follows. Nonetheless, it is of great interest to examine whether the distribution of inter-time changed with respect to the Tohoku-oki EQ. If we can identify exactly when the change of distribution occurred, this provides useful information on a possible precursor for the Tohoku-oki EQ. To shed light on this, we focused on the inter-time of LFEs with an epicenter in close proximity (<10 *km*) to the preceding LFE, which captures spatio-temporal correlations of consecutive LFEs. To identify homogeneous distributions, we performed cluster analyses on logarithms of LFE inter-time in a data-driven manner, which identified four homogeneous classes. Remarkably, examination of the relevance of these classes to the Tohoku-oki EQ suggests that the activity of LFEs in the shortest inter-time class (median 24 seconds) diminished significantly at least three months before the Tohoku-oki EQ, and that complete quiescence occurred in this class 30 days before the Tohoku-oki EQ (p-value = 0.00014). In contrast with LFEs, conventional earthquakes did not become inactive during the same period. Further statistical analysis implies that this class together with a similar class of volcanic tremor may have served as a precursor of the Tohoku-oki EQ. Here, we refer to ‘precursor’ as a phenomenon that does not cause a large earthquake, but serves as a useful indicator. We discuss a generative model in terms of non-homogenous Poisson processes for these classes of LFE in which the shortest inter-time class is characterized by a generalized gamma distribution with a product of shape parameters *vκ* = 1.54 in the domain of inter-time close to zero. Lastly, we give a possible geodetic interpretation for the relevance of LFEs to the Tohoku-oki EQ.

## Results

We considered LFEs that occurred along the volcanic front in northern Japan (latitude greater than 37°N; Fig. 1a). Some of their locations are close to active volcanoes, while others are not. A close relationship between LFEs and volcanic activity is anticipated, but is not clearly understood^25^. We pre-processed LFE data to obtain inter-times between consecutive events. We considered two types of datasets, taking into account proximity of consecutive LFEs. First, we evaluated inter-times of remote LFEs that are separated more than 10 km. Hereafter, we refer to this dataset as ‘Remote LFE’. Second, we evaluate inter-times of neighboring LFEs less than 10 km, referring to this dataset as ‘Neighbour LFE’ (see more details in data and pre-processing in section of Methods).

### Preliminary analysis

**Figure 2:**
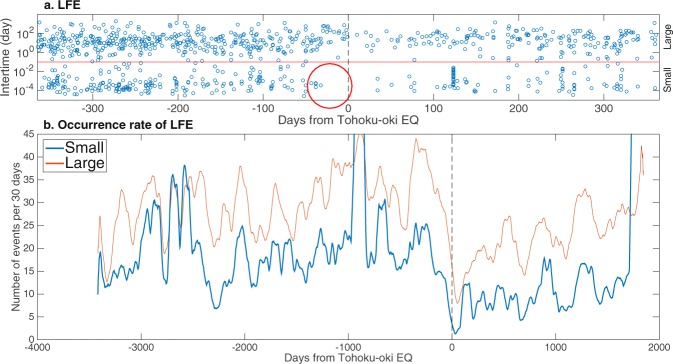
Preliminary analysis of the LFE occurrence patterns. Panel a: Each point denotes the pre-event of a pair of LFEs for a given inter-time. The horizontal axis denotes days from the Tohoku-oki EQ while the vertical axis denotes inter-time. The red line indicates a border between two classes: ‘Large’ class with inter-time greater than 10^−1^ days; ‘Small’ class with inter-time less than 10^−1^ days. Panel b: Evolution of LFEs for these two classes. The number of LFEs denoted by *n*(*z*) is evaluated with the width of window set 30 days backward: $n(z)={\sum }_{i=1}^{N}\,{\mathbb{I}}({t^{\prime} }_{i}\ge z-30){\mathbb{I}}({t^{\prime} }_{i}\le z)$ where ${\mathbb{I}}(\,\cdot \,)$ is an indicator function; ${t^{\prime} }_{i}={t}_{i}-{t}_{tohoku}$ with *t*_*tohoku*_ being the origin time of the Tohoku-oki EQ; *z* is an integer ranging from −3448 to 1847. Further, *n*(*z*) is smoothed using a moving average of a 90 day-window (45 days backward, 45 days forward). Roughly, ‘Small’ class here is naturally divided into two classes (S1 and S2) after the full-fledged analysis using the clustering technique (see Figs 3b and 5c).

As a preliminary analysis, we examined a time evolution of the inter-time distribution for Neighbour LFE (Fig. 2a). Remarkably, it was found that the number of occurrence of LFEs with small inter-time (less than 10^−1^ days) becomes null about 30 days before Tohoku-oki EQ. Motivated by this observation, we assumed two classes of LFEs, i.e., ‘Large’ class and ‘Small’ class, arbitrarily setting the cutoff value of inter-time to 10^−1^ days. For each class, we evaluated the occurrence rate with time-window of 30 days in the backward direction (Fig. 2b). It can be observed that, for both classes, the occurrence rate (smoothed by the moving average of ±45 days) had been decreasing for more than 100 days before Tohoku-oki EQ. In particular, the occurrence rate ‘Small’ class just before Tohoku-oki EQ became unprecedentedly small.

### Cluster analysis

**Figure 3:**
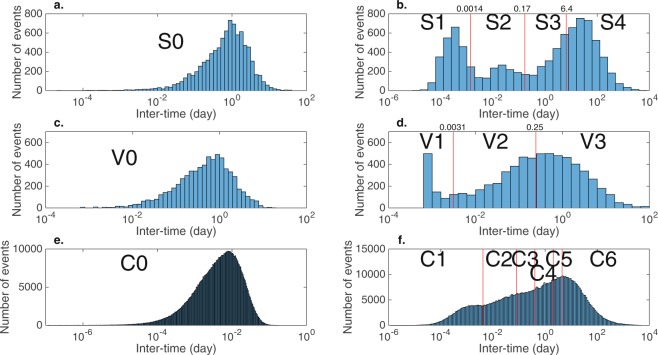
Distribution of inter-times between earthquakes. Panel a: Inter-time distribution of remote pairs of LFEs. Panel b: Inter-time distribution of neighboring pairs of LFEs. Red lines denote borders between four classes S1, S2, S3, and S4, which were yielded by fitting Gaussian mixture models to the logarithm of inter-time. Cutoff days for these borders are shown in the top of the panel. Panel c: Inter-time distribution of remote pairs of volcanic tremors. Here, we define ‘remote’ as different volcanoes. Panel d: Inter-time distribution of neighboring volcanic tremors, where the definition ‘neighboring’ denotes same volcano. Red lines denote borders between three classes V1, V2 and V3. Panel e and f: Inter-time distribution for conventional EQ.

The preliminary analysis suggests the importance of identifying classes of inter-time, which may reveal the underlying nature of LFEs, in particular, related to Tohoku-oki EQ. However, a visual inspection of the inter-time distribution (Fig. 3) shows that there are at least more than two classes. Because it is reasonable to expect that LFEs in different classes may be caused by different mechanisms thus giving rise to their characteristic behavior, it is of fundamental interest and of practical importance to identify homogeneous classes more rigorously. For this purpose, we carry out a cluster analysis for a multimodal distribution of the logarithm transformed inter-time with base 10 in the subsequent sections (see Methods for details).

### LFE

For remote LFEs, the inter-time ranges widely from 2.16 seconds to 17.1 days. It appears that the distribution of the logarithm of inter-time is unimodal with small skewness (Fig. 3a). Since the inter-distance between consecutive events in this dataset is more than 10 km, it is reasonable to assume that the direct causal relationship between consecutive events is relatively small. We did not carry out further analysis for this dataset, but keep it as a reference distribution (denoting this class of inter-time as S0, Table 1). For neighboring LFEs, it appears that the distribution of the logarithm of inter-time is multimodal, with three modes (Fig. 3b), which could suggest at least three underlying distributions for this dataset. Fitting Gaussian mixture models suggests four clusters. The estimates of means and variances for each Gaussian component are summarized in Table 1. Following a conventional procedure of mixture models, we clustered the logarithms of inter-time, yielding four classes. Here, classes S1–S4 are arranged in ascending order of means. Note that the optimal mixture model identified two distinct classes, S3 and S4, which initially appeared to comprise a single cluster. Notably, classes S2–S4 have similar variances close to 0.40, whereas class S1 has a considerably smaller variance, 0.17. One may characterize the inter-time scale of these classes as follows: seconds for S1, minutes for S2, days for S3, and months for S4 (column of ‘Median Inter-time’ in Table 1).

**Table 1:** Characteristic of each class of earthquake.

EQ Type	Data Type	Class	Median Inter-time	Sample Size	Gaussian distribution fitted to logarithm of inter-time			Seismicity Anomaly			
								Long-term anomaly		Short-term anomaly	
								ROC		Quiescent	
					Weight	Mean	Variance	Cutoff	AUC	Period	P-value
LFE	Remote	S0	18 hr	8263	1	−0.24	0.40				
	Neighbour	S1	24 sec	2081	0.27	−3.51	0.172	−76 day	0.83	32.7 days	1.4 × 10^−4***^
		S2	27 min	1336	0.15	−1.63	0.35	249 day	0.44	50.2 days	2.4 × 10^−3**^
		S3	2.0 day	1599	0.13	0.21	0.42	−53 day	0.72	11.9 days	2.0 × 10^−2*^
		S4	35 day	3168	0.45	1.42	0.44	−88 day	0.70	1.06 days	5.9 × 10^−1^
Volcanic Tremor	Remote	V0	13 hr	6713	1	−0.37	0.41				
	Neighbour	V1	1.0 min	810	0.13	<−2.51	NA	273 day	0.60	44.1 days	9.0 × 10^−4***^
		V2	1.5 hr	2514	0.27	−1.03	0.21	300 day	0.77	3.84 days	1.7 × 10^−1^
		V3	1.2 day	3388	0.53	0.02	0.40	300 day	0.85	1.12 days	3.4 × 10^−1^
Conv. EQ	Remote	C0	7.0 min	700698	1	−2.40	0.40				
	Neighbour	C1	1.4 min	98309	0.12	−3.02	0.35				
		C2	31 min	127240	0.15	−1.47	0.66				
		C3	4.4 hr	92386	0.16	−1.10	0.99				
		C4	23 hr	112692	0.23	0.32	1.00				
		C5	3.1 day	62310	0.22	0.65	0.81				
		C6	16 day	204456	0.13	0.99	0.31				

Weight, mean, and variances are based on Gaussian mixtures for logarithms (base 10) of inter-time. ROC cutoff is the optimal value of cutoff to split inter-times into two segments for ROC analysis. Quiescent period denotes the period between the origin time of the Tohoku-oki EQ and the time that the last event of corresponding class took place before the Tohoku-oki EQ. P-values were evaluated by fitting a left-truncated exponential distribution (truncated point is one day) to the data before the Tohoku-oki EQ, where the mean value of the distribution was estimated using a robust statistic, median/log2^55^. Asterisks denote level of significance of p-values: ***p < 0.001; **p < 0.01; *p < 0.05.

### Volcanic tremors and Conventional EQ

For purposes of comparison, we also included volcanic tremors and conventional earthquakes in our analysis. We considered volcanic tremors that occurred in five volcanoes along the volcanic front (between 37°–41°N, Fig. 1a) with sufficient inter-time sample size. First, we evaluated the inter-time between two volcanic tremors that occurred in different volcanoes. We define this dataset as ‘Remote volcanic tremors’. Second, we separately evaluated the inter-time for each volcano, which has the same effect as constraining the proximity between two events, just as among neighboring pairs of LFEs (‘Neighbour volcanic tremors’). For conventional earthquakes, we used the same definition of ‘remote’ and ‘neighbor’ as in LFE. We did not restrict conventional EQs based upon magnitude.

For remote volcanic tremors, the inter-time ranges widely from 60.0 seconds to 17.4 days. It appears that the distribution of the logarithm of inter-time is unimodal with small skewness (Fig. 3c). For neighboring volcanic tremors, we aimed to identify distinct classes of inter-time in a similar manner as with LFEs. First, we extracted samples V1 with very short inter-times, which do not seem to follow a Gaussian distribution. For the remaining samples, we applied Gaussian mixture models, which identified four optimal classes. Among these four, we focussed on two classes with mixture proportions (weight) greater than 0.05. The samples are subsequently allocated to one of these classes based on the conventional classification in mixture models^26^. As a consequence, we had three classes for inter-times of volcanic tremors V1, V2, and V3 (Fig. 3d, Table 1). As with LFEs, one may characterize inter-time scales of these classes as minutes for V1, hours for V2, and days for V3 (Table 1).

Note that we did not take into account possible overlaps of samples of inter-times between LFEs and volcanic tremors, which are negligible. However, this does not preclude interplay between LFEs and other volcanic phenomena, like tremors and EQ swarms. For instance, it is worth noting that more than 95% epicenters of class S2 during the bursting period [−950, −900] days were actually localized inside a narrow rectangular region of latitudes 43.34°N–43.44°N and longitudes 143.95°E–144.05°E in the vicinity of the Meakandake volcano. This burst preceded the singular tremors^27^ that started on day −893 (September 29, 2008) by 7 days. Further analysis of the characterization and intermittency of S2, based on such observations would be interesting.

For remote conventional EQs, inter-time ranges from 0.01 seconds to 4.26 hours. It appears that the distribution of the logarithm of inter-time is unimodal with small skewness (Fig. 3e). For neighboring conventional EQs, we carried out a cluster analysis to identify six clusters (Fig. 3f, Table 1). In contrast with the results for LFEs, the variance in each cluster varies considerably. Interestingly, however, the variance of class C0 has a similar value to that of class S0 (0.402 and 0.399, respectively). We will return to this point in the Discussion.

### Comparison of distributions

Distributions of inter-time have been empirically/theoretically studied for conventional EQs^21–24,28–30^, which suggest that its shape approximates a gamma distribution, but the exact distribution is not known. Currently, there is no consensus on the exact distribution of intertimes. In the present paper, we have fitted Gaussian mixture models to log-tramsformed inter-times. From the point of view of inter-time, this amounts to fitting a log-normal distribution to each class:

1 $$f(x) \sim \frac{1}{x\sqrt{{\sigma }^{2}}}\exp \{-\frac{{({\mathrm{log}}_{10}x-\mu )}^{2}}{2{\sigma }^{2}}\},$$

where *x* is inter-time, and *μ* and *σ*^2^ are the mean and variance of log-transformed inter-time. As can be seen in Eq. (1), the value of *σ*^2^ is scale-invariant with respect to data: through the transformation of $x\to ax$, the variance *σ*^2^ does not change. Hence, the characterization of a distribution by variance *σ*^2^ provides a useful tool to compare the shape of the distribution for data of different time-scales. Remarkably, the classes of LFE and volcanic tremors in our data are characterized by two specific values of *σ*^2^ (Table 1): Classes S0, S2, S3, S4, V0, and V3 have variances *σ*^2^ close to 0.4, whereas classes S1 and V2 have variance close to 0.2. This observation suggests that these two groups may have different underlying mechanisms of occurrence.

## Anomalies Related to the Tohoku-oki EQ

**Figure 4:**
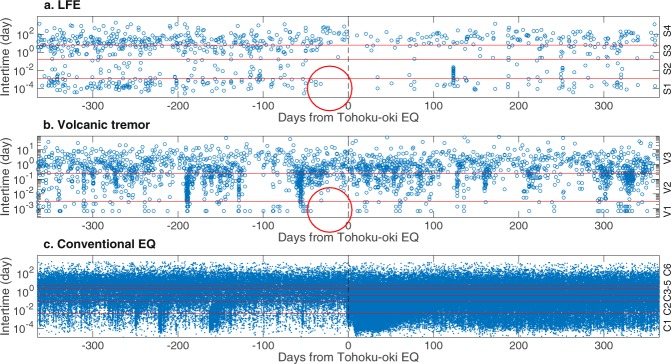
Raw data showing occurrence of neighboring events one year before and after the Tohoku-oki EQ. Panel a: LFE. Each point denotes the pre-event of a pair of LFEs for a given inter-time. The horizontal axis denotes days from the Tohoku-oki EQ while the vertical axis denotes inter-time. The red lines indicate borders between classes. LFEs of class S1 ceased before the Tohoku-oki EQ (red circle). Panels b and c: the same plots for volcanic tremors and conventional EQs.

Once our data-driven classification was performed, certain anomalous seismicities of LFEs and volcanic tremors became evident with respect to the timing of the Tohoku-oki EQ (Fig. 4). It appears that the seismicities of some classes of LFE and volcanic tremors changed with respect to the Tohoku-oki EQ. Importantly, such changes in S1 and V1 can be observed just before the Tohoku-oki EQ (red circle in Fig. 4). We investigated such anomalies, focusing on seismicity in each class from both long- and short-term perspectives. Here, long-term signifies several months while short-term indicates from several days to several weeks. For simplicity, we transformed the time of occurrence as $t^{\prime} =t-{t}_{tohoku}$ where *t* is the time of occurrence and *t*_*tohoku*_ is the origin time of the Tohoku-oki EQ.

### Long-term anomaly

**Figure 5:**
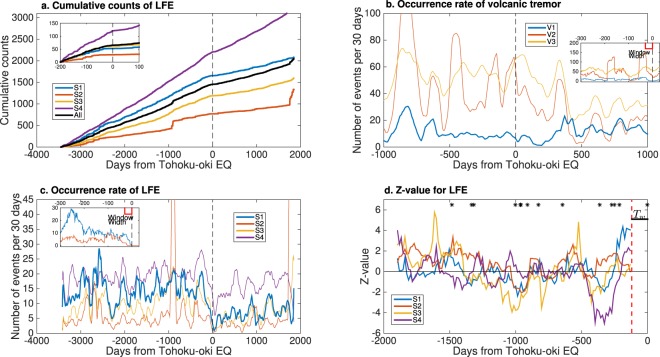
Processed data regarding the occurrence of neighboring events. Panel a: Evolution of cumulative LFEs for classes S1, S2, S3 and S4. The black curve denotes cumulative events for all classes divided by four. Note that the bursting behavior of S2 is observed in the period between −950 and −900 days and in the period between 1750 and 1850 days. In these periods, more than 95% of epicenters of S2 are localized in the rectangular area between latitudes 43.34°N–43.44°N and longitudes 143.95°E–144.05°E close to the Meakandake volcano. Panel b: Evolution of volcanic tremors for classes V1, V2 and V3. The number of volcanic tremor is evaluated in the same manner as in Panel c. Panel c: Evolution of LFEs for classes S1, S2, S3 and S4. The number of LFEs denoted by *n*(*z*) is evaluated with the width of window set 30 days backward: $n(z)={\sum }_{i=1}^{N}\,{\mathbb{I}}({t^{\prime} }_{i}\ge z-30){\mathbb{I}}({t^{\prime} }_{i}\le z)$ where ${\mathbb{I}}(\,\cdot \,)$ is an indicator function; ${t^{\prime} }_{i}={t}_{i}-{t}_{tohoku}$ with *t*_*tohoku*_ being the origin time of the Tohoku-oki EQ; *z* is an integer ranging from −3448 to 1847. For the main graph, *n*(*z*) is further smoothed using a moving average of a 90 day-window (45 days backward, 45 days forward) while it is not smoothed in the inset where occurrence rates for classes S1 and S2 are displayed. Panel d: Z-values for each class of LFE. We set the width of window (*T*_*w*_) to 120 days and the moving step to 14 days. The initial time (*t*_0_) is Jan. 1, 2006, while the terminal time (*t*_*e*_) is March 8, 2011. We did not consider spatial differences, but instead we used all LFE data for each class. On the top of the panel, the occurrence of large earthquakes with magnitude larger than 6 is denoted by black asterisks.

We examined a long-term anomaly, focusing on the evolution of seismicity in each class of LFE (Fig. 5a,c). A brief inspection of Fig. 5c suggests that the rate of occurrences decreased after the Tohoku-oki EQ for classes S1–S4. In particular, such a change was drastic for class S1, which can be observed as a ‘kink’ in the evolution of cumulative number of occurrence (Fig. 5a). Moreover, the decrease in rate of occurrence seems to have occurred some days before the Tohoku-oki EQ (Fig. 5c). We examined this hypothesis by means of ROC (Receiver Operating Characteristic) analysis^31^ (see section of ROC analysis for long term anomaly of Supplementary Materials), which yields an optimal cutoff day between large and small occurrence rates (Table 1). Remarkably, the optimal cutoff day for class S1 is −76 days, i.e., 76 days before the Tohoku-oki EQ, which quantitatively suggests that an anomalous behavior (quiescence) occurred 2.5 months before the Tohoku-oki EQ. This optimal cutoff day is robust to changes of the time scales to shorter periods, such as per week and per day, used in evaluating the occurrence rate, while the corresponding area under the curve (AUC) values tend to be smaller (Supplementary Table S1). The latter suggests that the long-term anomaly is most clearly captured if we use the occurrence rate per month. Similar analysis was performed for volcanic tremors (Fig. 5b, Table 1). In this case, however, the optimal cutoff day is positive, not capturing a long-term anomaly prior to the Tohoku-oki EQ.

The question then arose whether quiescence of class S1 could be detected if we use only data before the Tohoku-oki EQ. In terms of prediction, it is important to capture such a precursor before the earthquake. For this purpose, we evaluated ‘Z-value’^1,32^, which is useful for measuring seismic quiescence: A large value of Z provides evidence of seismic quiescence during a target period. Note that we did not consider spatial differences of seismicity in this analysis, but we used all data irrespective of the epicenters. The results of Z-values are displayed in Fig. 5d in which class S1 clearly shows quiescence approaching the Tohoku-oki EQ, while the remaining classes did not. In the beginning of 2010, the Z-value of class S1 was low, but it jumped over 2 when the window focuses on the target period between −229 days and −109 days (the width of window is 120 days), which shows that the Z-value analysis detected quiescence of class S1 earlier than in the AUC analysis (−76 days), i.e., at least three months before the Tohoku-oki EQ. After this period, the Z-value of class S1 remained as high as 4 just before the Tohoku-oki EQ. On the other hand, the Z-values of S2, S3 and S4 were not high, just before the Tohoku-oki EQ, which suggests that these classes do not show long-term anomalies preceding the Tohoku-oki EQ.

### Short-term anomaly

Next, we investigated the seismicity in more detail from a short-term perspective. It is of great interest to detect short-term anomalies, which might work as an immediate portent of the Tohoku-oki EQ.

**Figure 6:**
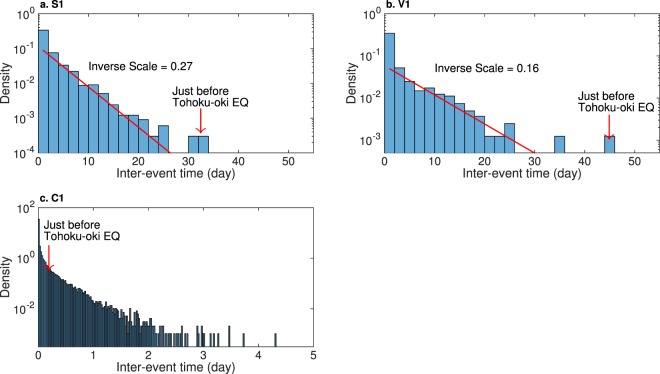
Distributions of inter-event time (density). Panel a for class S1. In this panel, we discarded inter-event times smaller than the cutoff interval for class S1. The red line was estimated by fitting a truncated exponential distribution with lower cutoff of 1 day where the mean was evaluated by a robust statistics with median/log(2), which is less influenced by outliers^55^. The estimated density function was normalized such that the sum of probability that inter-event time is greater than one day matches the empirical one. The slope of the line (inverse scale for exponential distribution) is displayed as text. Panel b: the same plot for class V1 of volcanic tremors. Panel c: the same plot for class C1 of conventional EQs. In this panel, we did not fit an exponential distribution.

Remarkably, a close observation of seismicity (Fig. 4a) suggests that the number of occurrences in class S1 and S2 became null about 30 days before the Tohoku-oki EQ. A similar anomaly is also observed for V1 (Fig. 4b). To statistically evaluate the anomaly of such quiescence, we focus on an inter-event time distribution within each class of LFE and volcanic tremors. To this end, we reformulated the inter-time of consecutive events for a given class label. We can expect that an event in each class occurs following Poisson process with sufficient time from the previous occurrence (Fig. 6a,b; but this is not the case for conventional EQs, Fig. 6c). Hence, we evaluate p-values of the length of null seismicity from the Tohoku-oki EQ (Table 1), based on an exponential distribution for each class. We arbitrarily truncated the fitted data to one day. For LFEs, the p-values in classes S1 and S2 were significant at 0.05 with Bonferroni correction^33^. On the other hand, for volcanic tremors it was significant only for class V1. In summary, these results suggest that classes S1, S2 and V1 may work as a short-term precursor for the Tohoku-oki EQ. In contrast, no anomalous quiescence was observed for conventional EQs (Figs 4c and 6c).

**Figure 7:**
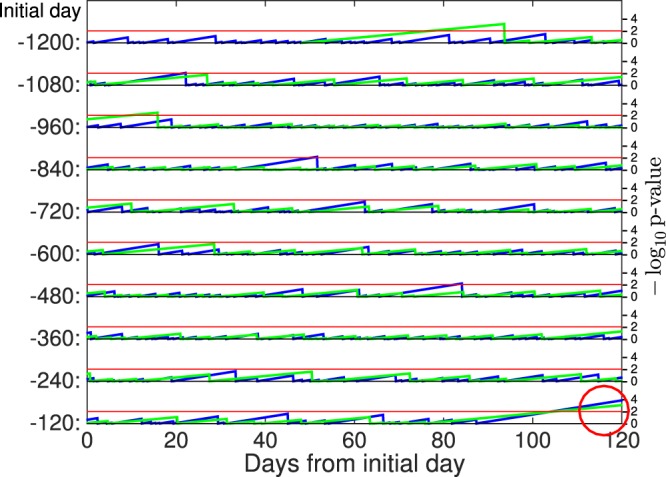
Evolution of p-values of inter-event time for class S1 (blue) and V1 (green), respectively. The horizontal axis denotes time (day) and the vertical axis negative logarithm of p-values with base 10. Here, time is evaluated as days from the Tohoku-oki EQ. Since volcanic tremors of class V1 are observed only after −1152 days, we consider the time span from −1200 days to 0 from the Tohoku-oki EQ, which is split into 10 sub-time spans. Each row in the plot denotes a particular sub-time span in which the initial day is shown at the left. P-values are evaluated using parameters in a fitted exponential distributions as detailed in the caption of Fig. 6, discarding inter-event times smaller than the cutoff inter-time that defines S1 and V1, respectively. The horizontal red line denotes the significance level 0.01 (the corresponding negative logarithm is 2). P-values of both class S1 and class V1 take four just before the Tohoku-oki EQ (red circle).

Further, we examined how significant these short-term anomalies are, compared with other periods of time. The inter-event time for both class S1 and V1 seems to follow an exponential distribution (Fig. 6a,b) except for the tails. However, the observed values of long inter-event times before the Tohoku-oki EQ do not seem to lie in the range of the corresponding exponential distribution. This suggests that such anomalous long quiescence just before the Tohoku-oki EQ may have been induced by mechanisms different from those at other periods of time. Note that such long inter-event time is not unique to the period just before the Tohoku-oki EQ. For class S1, a long inter-event time (longer than 30 days) was observed in mid August 2001 and early April 2005, and for class V1 in mid January 2008. Within two months of the onset of these quiescence, no earthquake larger than magnitude 7 occurred in northern Japan. However, the simultaneous quiescence of both S1 and V1 is unique to the period between Day −32 and Day 0 (the day of the Tohoku-oki EQ). To clarify this, we evaluated p-values of inter-event times in continuous time space, which is defined as the time from the latest event of class S1 (or, class V1), using the fitted exponential distribution. We found that observed p-values as low as 10^−3^ simultaneously for both classes S1 and V1 are unique to the Tohoku-oki EQ (Fig. 7). Simple calculation based on exponential distributions fitted to these classes suggests that such simultaneous quiescence of S1 and V1 is quite a rare event that could occur only once in 1300 years if we assume that events of these classes occur independently (for S1 once in 1/(0.27/day × 365 day × 0.00014) = 72 years; for V1 once in 1/(0.16/day × 365 day × 0.0009) = 19 years), where we used the inverse scales of the exponential distributions, 0.27/day for S1 and 0.16/day for V1, respectively (Fig. 6a,b). Certainly, this rough estimate should be considered with care since we have not taken into account the correlation of inter-event times between S1 and V1, which is beyond the scope of the present paper. Understanding such dependence between different classes could give us further insights into the underlying mechanisms of the LFEs and volcanic tremors; we leave it as an interesting open problem.

## Discussion

First, we discuss interpretations of both LFEs and conventional EQs in terms of fitting different types of probabilistic distributions. Let us assume that the occurrence of LFEs follows a non-homogeneous (compound) Poisson distribution^24,34^ conditional on the rate parameter *θ*, the distribution of inter-time *x* is given by an exponential distribution *θ* exp(−*θx*). Also, denoting weight for *θ* as *g*(*θ*) (probability density function), the marginal distribution of the inter-time can be in general expressed by^35^

2 $$f(x) \sim {\int }_{0}^{\infty }\,{\theta }^{2}\exp (\,-\,\theta x)g(\theta )d\theta \mathrm{.}$$

The probability density *g*(*θ*) can be modeled as a function of time *t* in the framework of Ohmori’s law^24,36^. However, in the case of LFEs, we have little evidence that the seismicity of LFEs follows Ohmori’s law (Supplementary Fig. S3). For simplicity, we do not parameterize *θ* with time *t*.

In the case of LFEs, our empirical data suggest that *f*(*x*) may follow a power law for S2–S4, but not for S1 (Supplementary Figs S4 and S5). For classes C0 and S0, we carry out more vigorous analysis, because full data are available for these classes. Classes C0 and S0 have similar variances in the logarithm of inter-time distribution (Table 1), but it is observed that these two classes may follow different generative models (Supplementary Fig. S6). We examine model-fitting to inter-time in these classes by two types of distributions: Lomax distribution (or, Pareto type II distribution)^37^

3 $$f(x)=\frac{\alpha {\beta }^{\alpha }}{{(x+\beta )}^{\alpha +1}}$$

and a generalized gamma distribution^22^, which may be followed by conventional EQs in a stationary period (but not in a period containing large main shocks^38^),

4 $$f(x)=\frac{\nu }{{\sigma }^{\nu \kappa }{\rm{\Gamma }}(\kappa )}{x}^{\nu \kappa -1}\,\exp \{\,-\,{(x/\sigma )}^{\nu }\},$$

where all relevant parameters are positive. For classes C0 and S0, a BIC-based model selection suggests a generalized gamma distribution. The exponent of *x* in Eq. (4), i.e., *vκ* − 1, takes values between −0.1 and 0.0 for both classes, which suggests that the tail of the distribution decays exponentially (Table 2). Regarding classes S1, S2, S3, and S4, full data are not available. The aforementioned model selection is not straightforward, because the method of fitting a truncated generalized gamma distribution is not well established. Hence, we consider an approach of matching variances of the logarithm of inter-time. It can be shown that the variance of log_10_*x* for a Lomax distribution in Eq. (3) is analytically given by $(\psi ^{\prime} (\alpha )+{\pi }^{2}/\mathrm{6)}/{(\mathrm{log}\mathrm{10)}}^{2}$ where $\psi ^{\prime} (\alpha )={\sum }_{k=0}^{\infty }\,1/{(k+\alpha )}^{2}$ is the first derivative of the digamma function with respect to *α*. Note that the variance is not related to *β* (because the variance of the logarithm of inter-time is invariant of scales of inter-time). Since $\psi ^{\prime} (\alpha )$ monotonously decreases, so does the variance with respect to *α* (Supplementary Fig. S7a), which converges to $({\pi }^{2}/6)/{(\mathrm{log}10)}^{2}\approx 0.310$ as *α* → ∞. It can be shown that a homogenous Poisson distribution corresponds the case of *α* → ∞. This analytical result can explain the variances of the logarithm of inter-time of S0, S2, S3, and S4 (also V0 and V3), suggesting the range of values of *α* between 1.9 and 5.2. In contrast with S0, S2, S3, and S4, the variance for class S1 (as well as V2) takes a much smaller value 0.17 (Table 1) than the minimum value 0.310 suggested by a Lomax distribution. This implies that the inter-time of class S1 (as well as V2) does not follow a Lomax distribution. Next, we consider a generalized gamma distribution. It can be shown that the variance of the logarithm of inter-time is given by $\psi ^{\prime} (\kappa )/{(\nu \mathrm{log}10)}^{2}$, which is in similar form to that of a Lomax, but without the additive term *π*^2^/6. It can be shown that the variance of the logarithm of inter-time can take those estimated variances of all classes including S1 and V2 by tuning *v* and *κ* (Supplementary Fig. S7b). These results suggest that inter-time in class S1 may follow a generalized gamma distribution rather than a Lomax distribution, while for classes S2, S3, S4 (also V0 and V3) we cannot draw a definitive conclusion on model selection.

**Table 2:** Results of fitting a generalized gamma distribution.

Class	Fitting Method	Parameters in generalized gamma dist.			
		*σ*	*v*	*κ*	*vκ*
S0	MLE	0.66	0.76	1.2	0.94
C0	MLE	0.53	0.70	1.4	0.97
C0M4	MLE	1.46	0.77	0.63	0.49
C0M4b2011	MLE	1.41	0.97	0.70	0.68
C0M4b2011	MLE + MM	1.50	1.03	0.67	0.69
S1	MLE + MM	0.62	0.98	1.6	1.54
Global EQ Catalog		1.58 ± 0.15	0.98 ± 0.05	0.68 ± 0.05	0.67

For purposes of comparison, we rescaled inter-time in each dataset by multiplying the mean rate of seismicity^22^. The estimates of parameters are based on maximum likelihood estimation (MLE), or the hybrid of MLE and moment matching (MM) as described in section of Discussion. Class C0M4 denotes a class of conventional earthquakes with *M*_*w*_ ≥ 4 of class C0, whereas class C0M4b2011 denotes conventional earthquakes that are obtained by restricting class C0M4 to before 2011 (hence, no influence of the 2011 Tohoku-oki EQ). The difference of parameters between C0M4 and C0M4b2011 reflects the change in seismicity after the Tohoku-oki EQ. As a reference, the results based on a global earthquake catalog^22^ are also displayed.

For class S1, we further estimate parameters in a generalized gamma distribution. Matching means, given by $(\mathrm{log}\,\sigma +\psi (\kappa ))/\,\mathrm{log}\,10$, and variances to the data reduces the degree of freedom of the parameters *σ*, *v* and *κ* to one. Using this constraint, we estimate these parameters based on the principle of maximization of likelihood^33^. The results of optimization suggest that *v* ≈ 0.98 and *κ* ≈ 1.6 (Table 2; Supplementary Fig. S8). The value of *v* close to one suggests that a gamma distribution may well fit the data. This result is similar to the case of conventional EQs with the lower cutoff magnitude ranging from 5 to 6.5 in global seismic catalogs^22^, and the case of conventional EQs in northern Japan before the Tohoku-oki EQ with the lower cutoff magnitude 4 (‘C0M4b2011’ in Table 2). Note that the parameter *v* after the Tohoku-oki EQ considerably changed from the parameter *v* before the Tohoku-oki EQ as is seen from the difference of *v* between ‘C0M4b2011’ and ‘C0M4’ (conventional EQ with cutoff magnitude 4 in the whole period in our study). In case of conventional EQs in global seismic catalogs, the value of *κ* is estimated to *κ* ≈ 0.68; hence, the exponent of inter-time in Eq. (4) becomes $\nu \kappa -1=-\,0.33\,(\, < \,0)$, suggesting high frequency of earthquakes in a short period of time. On the other hand, in class S1, the exponent becomes 0.54 (>0), suggesting the repulsive nature of occurrences between two events. This result reveals a fundamental difference in occurrence of events: In conventional EQs, soon after a pre-event, it is more likely that a post-event will occur. In the case of class S1, soon after a pre-event, there is a quiescent time before a post-event. One may wonder whether the inferred quiescent period for a post-event in class S1 may be attributed to a technical problem of detectability of consecutive events in a short period of time. We examined this issue by means of explicitly removing the problem of detectability. In Supplementary Fig. S8, it is observed that events with inter-times between 0.1 × 10^−3^ day (8 seconds) and 0.15 × 10^−3^ day (12 seconds) are most likely to occur. Now, we fit a left- and right-truncated generalized gamma distribution to the inter-time of class S1. We keep the same upper cutoff (i.e., the cutoff day 0.0014 between class S1 and class S2) as before, but we set the lower cutoff to 0.2 × 10^−3^ (17.2 seconds), which has the effect of removing inter-times less than the lower cutoff (i.e., removing four bins from the left in Supplementary Fig. S8). As a result of fitting, we obtained *v* = 1.02, *κ* = 1.48 and $\nu \kappa -1\approx 0.51$, which are similar to the case without the lower cutoff. This confirms the quiescent period for a post-event in class S1.

Second, we discuss interpretations of class S1 as a precursor to the Tohoku-oki EQ. Our analysis suggests that seismic quiescence of class S1 showed up at least three months before the Tohoku-oki EQ, and that subsequently this type of LFE completely disappeared 30 days before the Tohoku-oki EQ (Table 1). The timing of onset of seismic quiescence is consistent with the timing of the observed abnormal change of level and temperature of groundwater in Goyo-onsen, 155 km northwest of the epicenter of the Tohoku-oki EQ. At Goyo-onsen, an anomalous drop of water level and temperature began three months before the Tohoku-oki EQ^6^. Similarly, an anomalous increase of Radon concentration was observed in the Izu Peninsula, 460 km southwest of the epicenter, three months before the Tohoku-oki EQ^7^. Furthermore, the timing of the complete disappearance of class S1 is consistent with the timing of onset of the presumed nucleation process near the epicenter^4^. On the other hand, our observation of quiescence of class S1 can be contrasted with the observed quiescence of conventional EQs, which began 23 years before the Tohoku-oki EQ^1^. Quiescence of conventional EQs may work as a long-term indicator, whereas class S1 may play a key role as an immediate harbinger of a large earthquake.

Now, we explore a possible geodetic interpretation for the observed phenomenon in LFE. In general, seismicity reflects accumulation of strains^39^. Our results suggest that across much of Tohoku region, the accumulation of strain was reduced prior to the Tohoku-oki EQ. A possible explanation for this phenomenon may be reduced movement of the North American plate in which the Tohoku region lies. On the other hand, during the same period of time, it is reported that a nucleation process took place near the epicenter^4^, which suggests an increment of strain. It is worth noting that the nucleation process began 30 days before the Tohoku-oki EQ, which coincides with the onset of the complete quiescence of class S1. These seemingly contradictory phenomena may be explained by the asperity model, which assumes strong coupling of some areas of the plate interface in subduction zones^40^. Several studies suggest the existence of asperities off-shore of Tohoku^41,42^, which is located west of the epicenter. These asperities lie geographically between the area of LFEs in the present study and the area of the nucleation process^4^. Based on the asperity model, we offer a possible explanation as follows. Long before the Tohoku-oki EQ, the Pacific plate had continued to push the North American plate to the west, but by the time of the onset (30 days before the Tohoku-oki EQ), the asperities had become strongly coupled. Hence, the strain accumulated east of the asperities, i.e., near the epicenter. In contrast, the rate of strain accumulation became reduced west of the asperities, which could result in the quiescence of class S1.

Finally, we discuss four limitations of the present study. First, to analyze seismicity from the LFE data, missing data could be a statistical issue, potentially biasing the data analysis. In general, LFEs of low magnitude are less likely to be captured by the network of seismic meters than those of large magnitude. In the present study, we focused on the inter-time between two LFE events, assuming that inter-time and magnitude of LFE are independent. Second, we assume that data availability of LFE in the JMA catalog did not change during our study. There are two factors that may challenge this assumption. One factor is the possible improvement of detective capability of LFEs owing to technical advancement of seismic meters. The major change in this period is that some stations, including Sendai, located in the Tohoku region started to use an F-net seismic network^43^ to upgrade their detective capability for earthquakes on Oct.1, 2001^44^. Also, the weighting function for hypocenter location program was upgraded on the same day. Taking account of this change of detective capability, we used the data for LFEs and conventional EQs after October 1, 2001. We suppose improvement of further possible detective capability has little influence on the results in the present study. The other factor is effect of the Tohoku-oki EQ: A large number of aftershocks might have masked LFEs in seismic meters^45–47^. If this is true, we expect that not only LFEs, but also conventional EQs with small magnitude would have been masked. To examine this, we evaluated a cutoff value of magnitude that ensures the Gutenberg Richter law for conventional EQs in the region of our study. It is observed that the cutoff magnitude was *M*_*c*_ ≈ 0.5 before the Tohoku-oki EQ, but that it became larger thereafter (*M*_*c*_ ≈ 1.2). However, after 9 months the cutoff magnitude returned to its level prior to the Tohoku-oki EQ. This observation suggests that we may not have to take into account the masking effect at least 9 months after the Tohoku-oki EQ. In Fig. 5c, low activity of class S1 is still observed 9 months (270 days) after the Tohoku-oki EQ. We conclude that the long-term anomaly in S1 would be observable even if we discarded the period of aftershocks. Importantly, our analysis of the long-term anomaly based on Z-value does not use the data after the Tohoku-oki EQ; hence, the results on the long-term anomaly in Z-value are intact for the effect of the Tohoku-oki EQ. Similarly, regarding the short-term anomaly, our analysis does not use the data after the Tohoku-oki EQ, either. Further, we did not take into consideration of the *M*_*w*_ 7.3 foreshock, which occurred two days before near the epicenter of the main shock. The derived anomalies in the present paper showed up well before the time of the foreshock occurrence, hence we assume that there is little influence of the foreshock on our results. Hence, the results in the short-term anomaly are also intact for the effect of the Tohoku-oki EQ.

Third, we did not take into account spatial differences^20^ of LFEs in the present paper. For instance, patterns of occurrence rates of LFEs slightly differ between Hokkaido region (latitude larger than 41.3°) and Honshu region (latitude less than 41.3°), but the similar anomaly of quiescence is observed in both regions before Tohoku-oki EQ if we focus on class S1 (Supplementary Fig. S9). These apparent simultaneous anomalies in Hokkaido region and Tohoku region may be attributed to the huge length of the slipping fault, with which both regions can be considered relatively “close” to the main shock. It would be an interesting research direction to carry out more thorough spatial analysis based on the LFE classification, and in particular, to compare the timing of the onset of the anomalies identified in the present paper. Fourth, one may wonder whether the precursor event discussed in the present paper may be generalized to other large EQs. For this end, we examined a Molchan diagram, which is often used to test a prediction performance in a general framework, taking into account a wide range of prediction models with their various thresholds^24,48,49^. In our context, we considered a prediction of large EQ with magnitude *M*_*w*_ ≥ 6 in terms of differences in weekly occurrence rate of class S1. However, the results for both Hokkaido region and Tohoku region do not suggest a possible causal relationship between class S1 and large EQs (Supplementary Fig. S10). On the other hand, we also performed a simple statistical test to examine a possible casual relationship. We separately carried out a (paired) Wilcoxon signed-rank test on differences of weekly occurrence rates of class S1 between one week before and two weeks before a large EQ for Hokkaido region and Tohoku region for various lower bounds of magnitudes of large EQs (Supplementary Table S2). The test shows no significant relationship except, perhaps, the smallest p-value 0.08 for the lower bound *M*_*w*_ = 6, which is close to the significance level 0.05. It is not certain, only from these results, whether the precursor event discussed in the present paper may be directly generalized to other generic large EQs. Further refinement of the framework, for instance, based on some classification, other than that by their magnitudes, of large EQs would be interesting to detect such associations, but it would require more data. Further research with some more ingenuity is required to clarify these points.

## Methods

In this section, we provide details on data, pre-processing, cluster analysis, ROC curve analysis, and Z-values, which were employed to yield the results in the present paper.

### Data

All data in the preset study, including LFEs, conventional EQs, and volcanic tremors, were obtained from JMA catalogs (Japan Meteorological Agency)^50^. We used 8263 LFEs that are flagged in the seismic catalog in the period from October 1, 2001 to March 31, 2016 (i.e., −3448 to 1847 days from the Tohoku-oki EQ). We consider LFE only after October 1, 2001, because detective capability of LFE by JMA considerably improved after this date. For conventional EQs, we focused on the same time period. For volcanic tremors, we considered volcanos with latitudes between 37.5°N and 42°N covering a wide area of northern Japan, and with more than 100 volcanic tremors. Six volcanoes met these criteria, but in volcano Zaozan (38°08′37″N, 140°26′24″E), the first volcanic tremor was not recorded before 300 days from the Tohoku-oki EQ. Due to this limited time period, we excluded this volcano for analysis. As a result, we included the following five volcanos in our analysis: Esan (41°48′17″N, 141°09′58″E), Iwatesan (39°51′09″N, 141°00′04″E), Azumayama (37°44′07″N, 140°14′40″E), Adatarayama (37°37′59″N, 140°16′59″E) and Bandaisan (37°36′04″N, 140°04′20″E)^51^ with observed volcanic tremors 183, 1306, 2733, 139 and 700, respectively. The time of observed volcanic tremors was: from −1165 day to 1108 day for Esan; from −1164 day to 1107 day for Iwatesan; from −1132 day to 1094 day for Azumayama; from −1123 day to 1028 day for Adatarayama; from −1161 day to 1112 day for Bandaisan. Here, we set the origin time of the Tohoku-oki EQ to 0 day.

### Pre-processing

Given a time series of EQ events, the inter-time of two events is simply defined as the time elapsed between two consecutive events. Here, we further extend this definition, taking into account the proximity of two events, as follows. First, the time of event occurrence is sorted in ascending order, *t*_1_ < *t*_2_ < … < *t*_*N*_, where *t*_*i*_ denotes the time (day) of occurrence of *i*th event; *N* sample size. Let Δ_*i*,*j*_ be the difference of time between events *i* and *j* (i < *j*), defined by Δ_*i*,*j*_ = *t*_*j*_ − *t*_*i*_. Denoting as *d*_*i*,*j*_ the distance (km) between epicenters of events *i* and *j*, we define the inter-time constrained by *d*_*i*,*j*_ as follows:

$${{\rm{\Delta }}^{\prime} }_{i,j}({d}_{min},{d}_{max})={{\rm{\Delta }}}_{i,j}{\mathbb{I}}({d}_{i,j} > {d}_{min}){\mathbb{I}}({d}_{i,j} < {d}_{max}),$$

where ${\mathbb{I}}(a)$ is an indicator function. This function simply sets inter-time to zero if the distance between events *i* and *j* is less than *d*_*min*_ or larger than *d*_*max*_, thus degenerating inter-time in such cases. Using this quantity, we generate a vector of inter-time denoted by Δ(*d*_*min*_, *d*_*max*_), which consists of *N* elements Δ_*i*_ for *i*th event as follows:

5 $${{\rm{\Delta }}}_{i}({d}_{{\min }},{d}_{{\max }})=\mathop{\min }\limits_{i < j,{\Delta ^{\prime} }_{i,j} > 0}\,{{\rm{\Delta }}^{\prime} }_{i,j}({d}_{{\min }},{d}_{{\max }}).$$

In a nutshell, Δ(*d*_*min*_, *d*_*max*_) represents a collection of inter-time in a specific range of distance between *d*_*min*_ and *d*_*max*_, where we allocate the inter-time to the pre-event, rather than the post-event.

With these notations, the dataset Δ(0, ∞) represents a collection of inter-times without any constraints on distance. In our data, the distribution of this type of inter-time is displayed in Supplementary Fig. S2a. Remarkably, it is observed that the distribution of inter-time differs between the upper part and the lower part, which are separated by the line of distance 10 km. This suggests that there are two heterogenous groups of inter-time, characterized by the inter-distance between consecutive events.

For a better understanding of the underlying mechanism of LFEs, it would be useful to analyze these groups separately. Note, however, that the upper and the lower part are closely intertwined in this dataset. To minimize such interactions, we set the cutoff point to 10 km to generate two datasets: Δ(10, ∞), referred to as ‘Remote LFE’, and Δ(0, 10) as ‘Neighbour LFE’ (Supplementary Fig. S2b), both of these having the sample size *N*. From the definition in Eq. (5), Remote LFE represents the inter-time of remote pairs of events (remote inter-time, with inter-distance larger than 10 km), while Neighbour LFE the inter-time of neighboring pairs (neighboring inter-time, with inter-distance smaller than 10 km).

### Cluster analysis

To estimate the underlying distribution for a given dataset, we fitted Gaussian mixture models^26^. In this model, a distribution of data, denoted as *f*(*x*), is given by summation of Gaussian distributions:

$$f(x)=\sum _{k=1}^{K}\,{w}_{k}\,\times \,{\rm{Gauss}}\,(x|{\mu }_{k},{\sigma }_{k}^{2}),$$

where *K* is the (estimated) number of classes; *w*_*k*_ is weight for the *k* th component; ${\rm{Gauss}}\,(\,\cdot \,|\mu ,{\sigma }^{2})$ is a Gaussian distribution for the *k* th component with mean *μ*_*k*_ and variances ${\sigma }_{k}^{2}$. Importantly, we can classify data points by allocating each data point to the most plausible component in this mixture model. Further, we manipulated the value of *K* from one to five. To select an optimal model (the value of *K* in the present case), the most popular criteria are the AIC (Akaike Infromation Criterion)^52^ and the BIC (Bayesian Information Criterion)^53^. It is well known that AIC is not consistent (i.e., the probability of identifying the true model is not necessarily one as the sample size goes to infinity), while BIC is consistent^54^. In our dataset, the sample size is relatively large (in the order of 1000). We accordingly used BIC for model selection in the present study.

### ROC curve analysis

Suppose we have *n* pairs of data (*x*_*i*_, *y*_*i*_) (1 ≤ *i* ≤ *n*) where *x*_*i*_ is numerical and *y*_*i*_ is binary. Now, let us assume that there is an unknown association between *x*_*i*_ and *y*_*i*_. We considered classifying these data points using only information on *x* and evaluate its performance, referring to the true label *y*. Here, our classifier was a binary classifier achieved by setting a value *x*_0_: if *x*_*i*_ < *x*_0_, we allocate *x*_*i*_ to one group, otherwise to the other group. Our question was, “to what extent this classifier can reveal the true label?”. To answer this question, we drew a ROC curve by manipulating a value of *x*_0_: a ROC curve represents a graphical plot of sensitivity verse (1-specificity) where sensitivity is defined as the number of true positive/(the number of true positive + the number of false negative); specificity is the number of true negative/(the number of true negative + the number of false positive). In this plot, the horizontal axis is (1-specificity) while the vertical axis is sensitivity. AUC represents the area surrounded by the ROC curve, the horizontal axis and the vertical line that passes through (1, 1). AUC takes a value between 0 and 1. A close value of AUC to one suggests a *x*-based classifier can yield the true label *y*.

### Z-value

‘Z-value’ evaluates seismicity in a target period against a background period, which is defined as

$$Z=({R}_{bg}-{R}_{w})/{({S}_{bg}/{n}_{bg}+{S}_{w}/{n}_{w})}^{1/2},$$

where *R*_*bg*_ and *R*_*w*_ are mean seismicity in the background period and the target period, respectively; *S*_*bg*_ and *S*_*w*_ are variances in the corresponding periods; *n*_*bg*_ and *n*_*w*_ the number of bins in the corresponding periods. In a nutshell, Z-value denotes the normalized difference of seismicity between the background period and the target period. In the present paper, we divided the whole period of observations from Jan. 1, 2006 to March 8, 2011 into bins of 14 days. We set the width of window (*T*_*w*_) for a target period to 120 days with a moving step of 14 days. Note that a background period is defined as a set difference between the whole period and a target period. In this setting, we counted the number of events, evaluating seismicity in each bin. Using seismicity in a bin, we evaluated means and variances in the target and the background periods, which led to the Z-value.

## Supplementary information

Supplementary materials

## Data Availability

All data used in this study are available from JMA catalogs (Japan Meteorological Agency, https://www.jma.go.jp/jma/indexe.html).
