# Supplementary material for: Classes of low-frequency earthquakes based on inter-time distribution reveal a precursor event for the 2011 Great Tohoku Earthquake
Source: Sci Rep. 2019 Jun 27;9:9330. doi: 10.1038/s41598-019-45765-0 (PMC6597560; doi:10.1038/s41598-019-45765-0)
Supplement: Supplementary file 1 — Supplementary materials [file 41598_2019_45765_MOESM1_ESM.pdf]

Supplementary Information for "Classes of low-frequency earthquakes based on inter-time distribution reveal a precursor event for the 2011 great Tohoku Earthquake"

Tomoki Tokuda[1,*] **and Hirohiko Shimada**[1,2]

[1]Okinawa Institute of Science and Technology Graduate University, Onna, Okinawa, 904-0495, JAPAN
[2]Department of Integrated Science and Technology, National Institute of Technology, Tsuyama College, 624-1 Numa, Tsuyama, Okayama 708-8509, JAPAN
[*]tomoki.tokuda@oist.jp

ROC analysis for long-term anomaly

ROC (Receiver Operating Characteristic) analysis has been widely used for decision-making in the domains of medical research and machine learning community (see section of ROC analysis in the Methods of the main text). To apply this analysis to our context, we manipulated the cutoff day from -300 to 300 days (we assume the cutoff day takes only integers), which splits the data points into two groups: a before-group (the time of occurrence is less than the cutoff day) and an after-group (the time of occurrence is larger than the cutoff day). This procedure yielded a binary label for each data point, showing the group to which the data point belonged. Using this labeling for each cutoff day, we evaluated the area under the curve (AUC) of the number of occurrences based on a logistic regression analysis. Finally, we evaluated the maximum AUC and the corresponding cutoff day (Table 1 in the main text). For instance, the optimal cutoff day for class S1 is -76 day, which means that the optimal cutoff point is on 76 days before Tohoku-oki EQ. That is to say, with this cutoff day, the overlap of occurrence rates in two group, i.e., one group (less than cutoff day), and the other group (greater than the cutoff day), becomes the smallest (Supplementary Fig. S1). It is found that class S1 gives the largest value of AUC (0.83), followed by class S3 (0.72), class S4 (0.70), and class S2 (0.44) if the occurrence rate is evaluated by the number of events per month (30 days). This suggests that the occurrence rate changed most in S1.

Table S1. Long-term anomaly of occurrence rate per week and per day. ROC cutoffs and AUC values were estimated in the same way as in Table 1 in the main text, which summarizes these quantities for occurrence rate per month.

	Occurrence rate per week		Occurrence rate per day	
Class	ROC Cutoff	AUC	ROC Cutoff	AUC
S1	-83 day	0.68	-88 day	0.56
S2	221 day	0.47	221 day	0.49
S3	-64 day	0.63	-67 day	0.55
S4	-100 day	0.62	-20 day	0.53

Table S2. Prediction performance of large EQs by occurrence rate of class S1. Prediction is based on whether the weekly occurrence rate of class S1 decreases. We took the base time point when a particular large EQ occurred, and evaluated the weekly occurrence rate twice: one from two weeks to one week before the large EQ; the other from one week before to the base time point. P-values are based on a (paired) Wilcoxon signed-rank test, which evaluates the difference between these weekly occurrence rates prior to the large EQ. If the negative difference (decrease case) is observed for a number of large EQs, it results in a small p-value. For this analysis, we used data obtained by three months before Tohoku-oki EQ, focusing on large EQs that occurred with longitude grater than 142°. Further, we discarded large EQs that occurred within one month of the preceding one. We manipulated a lower bound of large EQs from magnitude 5.0 to 7.0 by steps of 0.5, and separately performed the statistical test for Hokkaido region (latitude larger than 41.3°) and Tohoku region (latitude smaller than 41.3°).

	Threshold of magnitude	5.0	5.5	6.0	6.5	7.0
Hokkaido region	P-value	0.77	0.51	0.50	0.14	0.27
	Number of events	# 11	# 35	# 26	# 11	# 7
Tohoku region	P-value	0.65	0.30	0.08	0.63	1.00
	Number of events	# 33	# 24	# 9	# 4	# 2

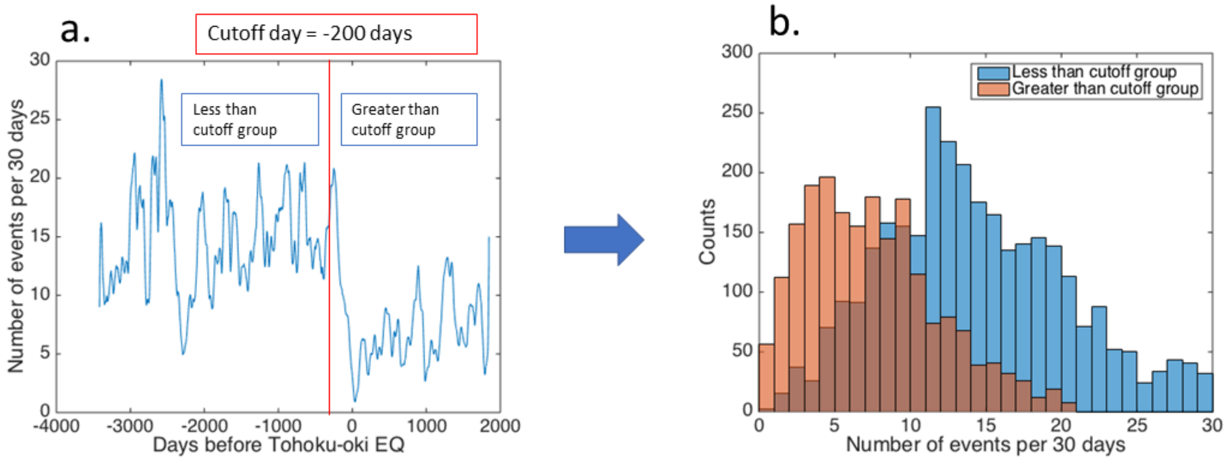

Figure S1. Illustration of application of ROC curve analysis to determine an optimal cutoff day. In this example, we set the cutoff day to -200 days. First, we separate data points into two groups: less than cutoff group and greater than cutoff group (Panel a). For each group, we plot a histogram of occurrence rates (Panel b). We subsequently evaluate the extent of overlapping of two groups by means of AUC in ROC curve analysis. For different values of cutoff day, we repeat this procedure, yielding a AUC value for each cutoff day. Finally, we estimate the optimal cutoff day that maximizes AUC, which corresponds to minimum overlapping.

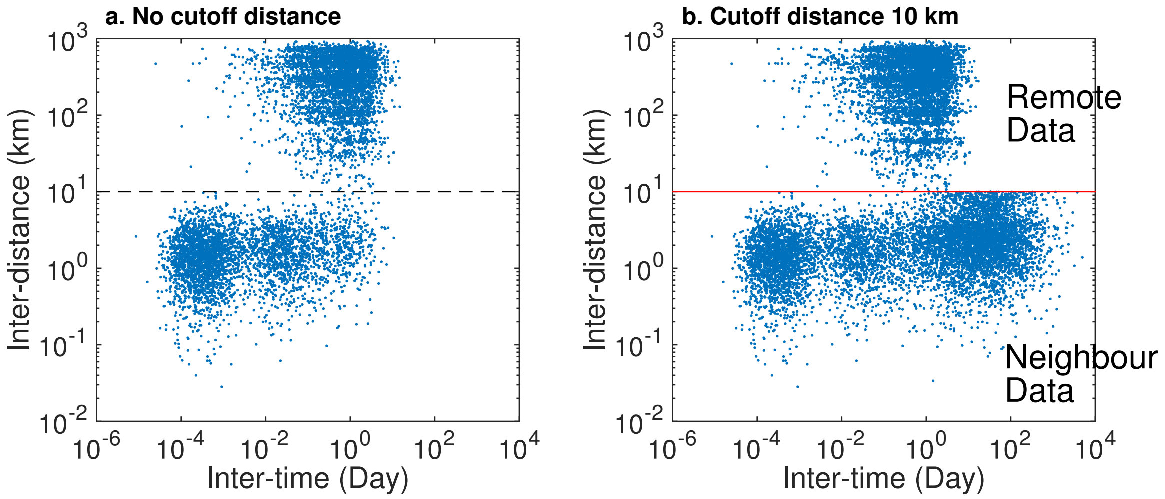

Figure S2. Distribution of LFE as a function of inter-time and inter-distance. Panel a: No inter-distance cutoff (dataset $\Delta(0,\infty)$). Panel b: With inter-distance cutoff 10 km. Dataset for remote pairs of events ($\Delta(10,\infty)$) in the upper part, and for neighboring pairs ($\Delta(0,10)$) in the lower part. The red line (inter-time distance 10 km) denotes the boundary between two datasets.

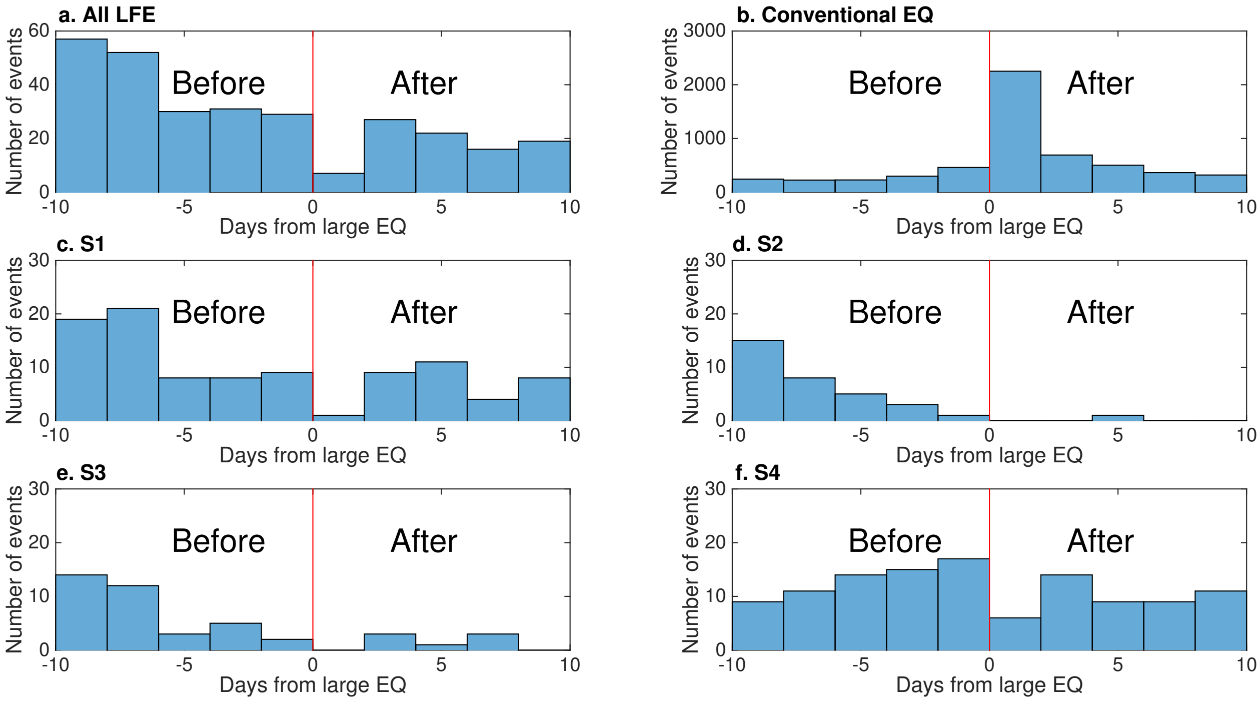

Figure S3. The number of events before and after 61 large earthquakes with $M_w \geq 5$. Panels a-f are for all LFE, conventional EQ, class S1, Class S2, Class S3 and Class 4, respectively. In this analysis, we focussed on the time period between Oct.1, 2001 and Feb.11, 2011 (30 days before the Tohoku-oki EQ), restricting areas: Latitude between 39°N and 41°N; longitude between 139°E and 145°E. For conventional EQ, we counted the number of events with $M_w \geq 2$. P-values of chi-square test are evaluated for differences of number of events between before and after the large earthquakes: 1.2×10^{-10} for all LFE; 4.6×10^{-274} for conventional EQ; 6.6×10^{-4} for class S1; 3.5×10^{-8} for class S2; 5.1×10^{-6} for class S3; 0.07 for class S4. Except class S4, the difference of seismicity between before and after large earthquakes are significant at 0.05 level. In case of conventional EQ, the detected difference can be understood as the tendency of high seismicity after large earthquakes, while in case of classes S1, S2, and S3 as the tendency of low seismicity after large earthquakes.

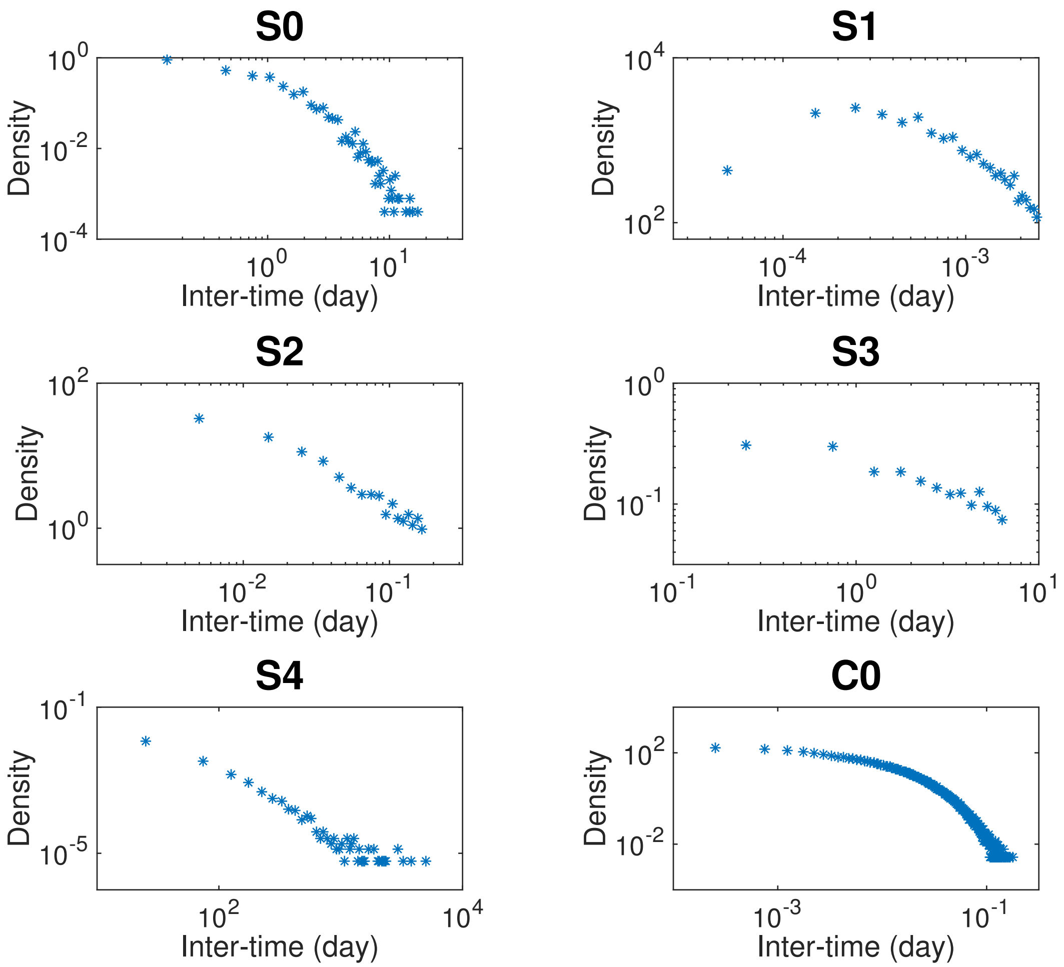

Figure S4. Log-log plots of inter-time and probability density for classes S0-S4 and C0.

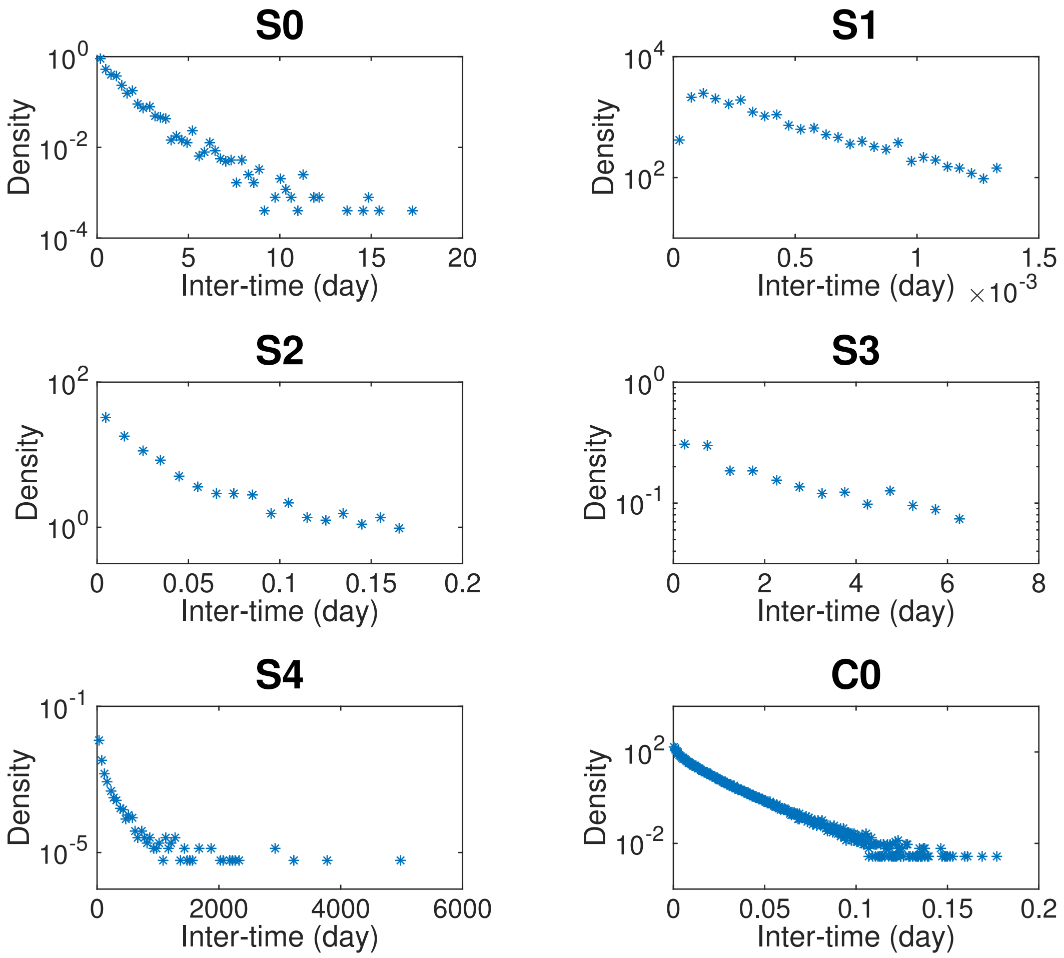

Figure S5. Semilog plots of inter-time and probability density for classes S0-S4 and C0.

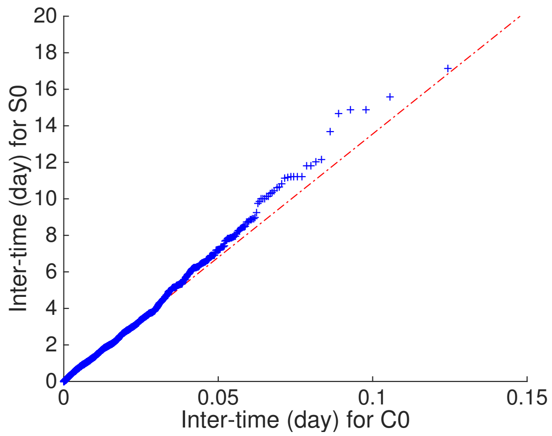

Figure S6. The qq-plot[1] of inter-time distributions between class C0 and S0. Each dot denotes a particular quantile for both distributions of class C0 and class S0. If the shape of density distributions is the same between two, these dots are supposed to lie in the red line. The difference of the distributions may be attributed to different shape parameters ν and κ in a generalized gamma distribution (Table 2 in the main text).

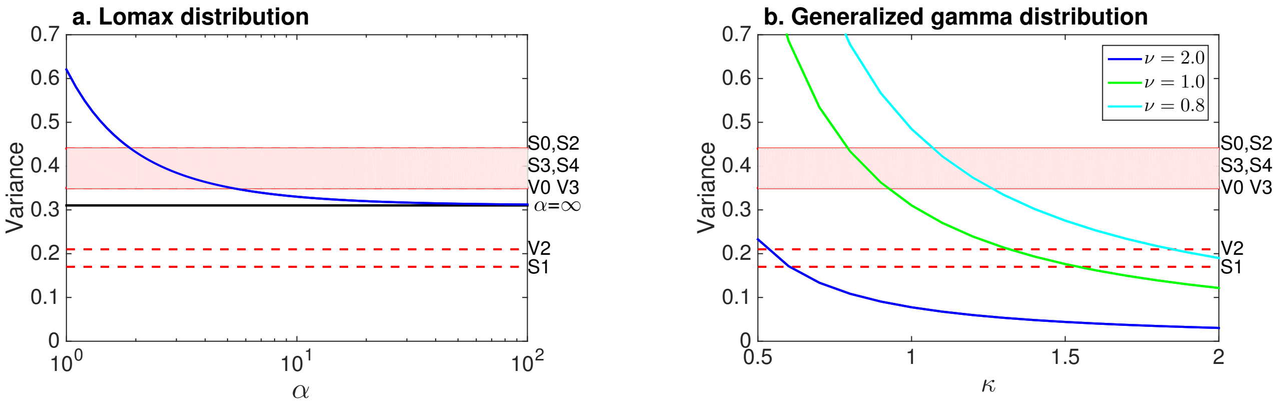

Figure S7. Theoretical values of variances for the logarithm of inter-time as a function of a given parameter to a specific distriubtion. Panel a: For Lomax distribution. The horizontal axis denotes the parameter α of Eq.(3) in the main text, while the vertical axis the variance of the logarithm of inter-time. The red area denotes the range of estimated variances of class S0, S2, S3, S4, V0, and V3 (Table 1 in the main text), while the dashed line denotes the estimate variances of class V2 and S1. The black line denotes the asymptotic value of variances as $\alpha \to \infty$. Panel b: For a generalized gamma distribution. The horizontal axis denotes κ in Eq.(4) in the main text. We manipulated the parameter ν to 0.8, 1 and 2. It can be shown that for fixed value ν, the variance converges to 0 as $\kappa \to \infty$.

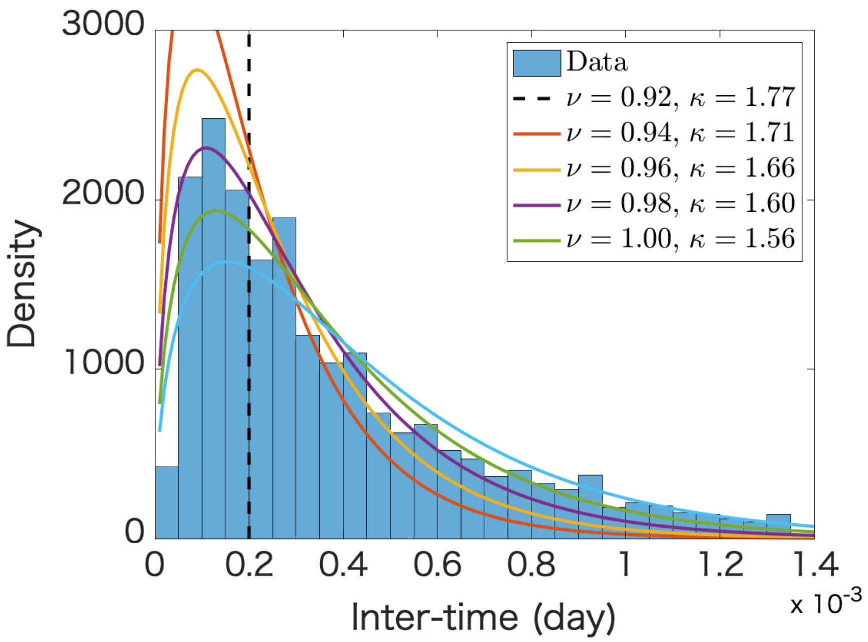

Figure S8. Density functions of inter-time of class S1. We fitted a generalized gamma distribution to the data, imposing the constraint that the mean and the variance of the logarithm of inter-time should match those estimated values of class S1, -3.51 and 0.172 (Table 1 in the main text), respectively. With this constraint, the triple of parameters σ, ν, and κ in a generalized gamma distribution has one degree of freedom. We manipulated ν while evaluating σ and κ using the constraint of the mean and the variance. The dashed black line denotes the lower cutoff value 0.2×10^{-3} of inter-time, by which we truncated the data for re-fitting a generalized gamma distribution as a consistency check.

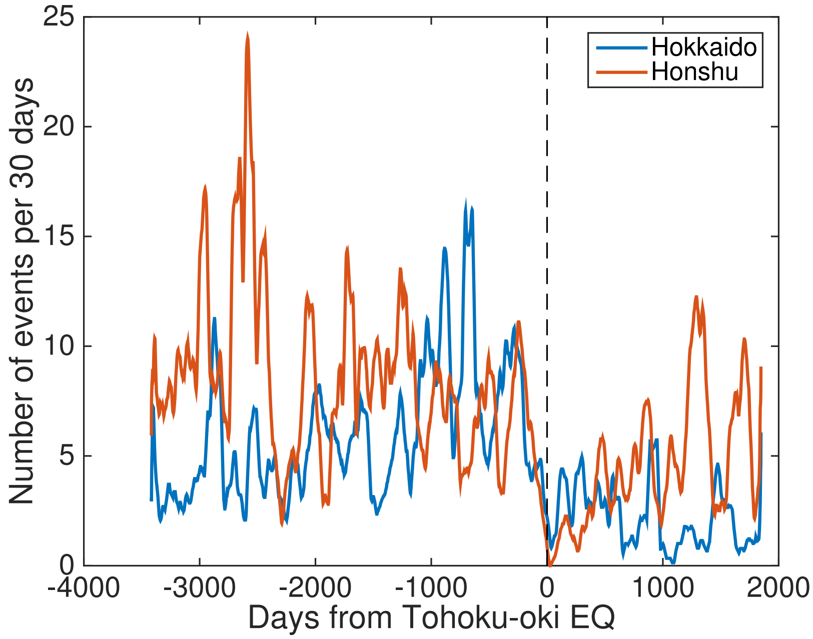

Figure S9. Evolution of occurrence rate of class S1 for Hokkaido region (latitude larger than 41.3°) and Honshu region (latitude less than 41.3°). We used the same setting for estimation of occurrence rate and smoothing of the graph as in Fig.5c of the main text.

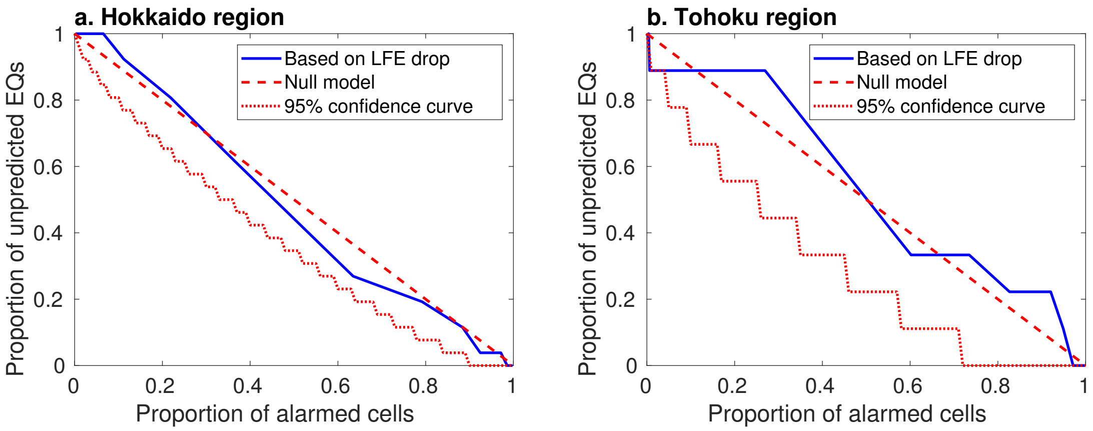

Figure S10. Molchan diagrams based on changes in occurrence rate of LFEs per week before large EQ with magnitude $M_w \geq 6$. Panel a: Hokkaido region (latitude larger than 41.3°); Panel b: Tohoku region (latitude less than 41.3°). For these diagrams, we focussed on LFEs and large EQs that occurred more than three months before Tohoku-oki EQ, which were separately used for each region. For large EQs, we considered those with longitude grater than 142° in which most of large EQs occurred off-shore. Also, we discarded large EQs that occurred within one month of the precedent large EQ. Using these data, we came up with a Molchan diagram as follows. First, we segmented the target period by seven days, and evaluated occurrence rates of LFEs in each cell (segment). Second, we evaluated changes in occurrence rate of LFEs between two consecutive cells. Third, for each large EQ, we identified the closest cell that preceded in time (hence, pairs of a large EQ and a cell). Fourth, based on these pairs of a large EQ and a cell, we developed a prediction model for a large EQ. Setting a threshold of drop in occurrence rate of LFE, we evaluated the true positive rate (TPR), which is reflected in the vertical axis of a panel as (1-TPR). On the other hand, the horizontal axis denotes a proportion of alarmed cells. We manipulated the threshold covering all feasible drops. The blue bold line denotes a relationship between these two quantities based on our prediction model, while the red dashed line denotes the relationship based on the null model; the red dotted line denotes the 95% confidence curve. Note that due to the discrete nature of binomial distribution, the 95% confidence curve is not smooth.

References

1. Rice, J. *Mathematical statistics and data analysis* (Nelson Education, 2006).
